# Viral and host factors associated with SARS-CoV-2 disease severity in Georgia, USA

**DOI:** 10.1371/journal.pone.0317972

**Published:** 2025-04-01

**Authors:** Ludy R. Carmola, Allison Dorothy Roebling, Dara Khosravi, Rose M. Langsjoen, Andrei Bombin, Bri Bixler, Alex Reid, Cara Chen, Ethan Wang, Yang Lu, Ziduo Zheng, Rebecca Zhang, Phuong-Vi Nguyen, Robert A. Arthur, Eric Fitts, Dalia Arafat Gulick, Dustin Higginbotham, Azmain Taz, Alaa Ahmed, John Hunter Crumpler, Colleen Kraft, Wilbur A. Lam, Ahmed Babiker, Jesse J. Waggoner, Kyle P. Openo, Laura M. Johnson, Adrianna Westbrook, Anne Piantadosi

**Affiliations:** 1 Department of Pathology and Laboratory Medicine, Emory University School of Medicine, Atlanta, Georgia, United States of America; 2 Georgia Department of Health, Georgia Emerging Infections Program, Atlanta, Georgia, United States of America; 3 Atlanta Veterans Affairs Medical Center, Decatur, Georgia, United States of America; 4 Department of Medicine, Division of Infectious Diseases, Emory University School of Medicine, Atlanta, Georgia, United States of America; 5 Graduate Program in Genetics and Molecular Biology, Emory University, Atlanta, Georgia, United States of America; 6 Department of Biostatistics and Bioinformatics, Rollins School of Public Health, Emory University, Atlanta, Georgia, United States of America; 7 Emory Integrated Computational Core, Emory University School of Medicine, Atlanta, Georgia, United States of America; 8 Georgia Clinical & Translational Science Alliance, Emory University School of Medicine, Atlanta, Georgia, United States of America; 9 Emory Integrated Genomics Core, Emory University School of Medicine, Atlanta, Georgia, United States of America; 10 The Atlanta Center for Microsystems-Engineered Point-of-Care Technologies, Atlanta, Georgia, United States of America; 11 Department of Pediatrics, Emory University School of Medicine, Atlanta, Georgia, United States of America,; 12 Aflac Cancer and Blood Disorders Center at Children’s Healthcare of Atlanta, Atlanta, Georgia, United States of America; 13 Wallace H. Coulter Department of Biomedical Engineering, Emory University and Georgia Institute of Technology, Atlanta, Georgia, United States of America; 14 Department of Pediatrics, Pediatric Biostatistics Core, School of Medicine, Emory University, Atlanta, Georgia, United States of America; Yale University School of Medicine, UNITED STATES OF AMERICA

## Abstract

While SARS-CoV-2 vaccines have shown strong efficacy, the continued emergence of new viral variants raises concerns about the ongoing and future public health impact of COVID-19, especially in locations with suboptimal vaccination uptake. We investigated viral and host factors, including vaccination status, that were associated with SARS-CoV-2 disease severity in a setting with low vaccination rates. We analyzed clinical and demographic data from 1,957 individuals in the state of Georgia, USA, coupled with viral genome sequencing from 1,185 samples. We found no specific mutations associated with disease severity. Compared to those who were unvaccinated, vaccinated individuals experienced less severe SARS-CoV-2 disease, and the effect was similar for both variants. Vaccination within the prior 3-9 months was associated with decreased odds of moderate disease, severe disease, and death. Older age and underlying health conditions, especially immunosuppression and renal disease, were associated with increased disease severity. Overall, this study provides insights into the impact of vaccination status, variants/mutations, and clinical factors on disease severity in SARS-CoV-2 infection when vaccination rates are low. Understanding these associations will help refine and reinforce messaging around the crucial importance of vaccination in mitigating the severity of SARS-CoV-2 disease.

## Introduction

Vaccinations against SARS-CoV-2 have undeniably demonstrated high efficacy in preventing COVID-19 infections and improving disease outcomes [1–3], but their impact is challenged by the emergence of new variants carrying immune evasion mutations and the waning of immune responses over time [4]. Partly due to these issues, public trust in COVID-19 vaccines has diminished with each round of booster recommendations, especially in the southeast United States (US) [5]. Throughout the SARS-CoV-2 pandemic, the state of Georgia has consistently held one of the lowest rates of vaccine coverage in the nation [6]. One important approach to addressing vaccine hesitancy is to conduct studies – and disseminate their results – to populations with low vaccine uptake.

Understanding the impact of vaccination on SARS-CoV-2 disease outcomes requires consideration of other host factors such as underlying health conditions, sex, race, and socioeconomic factors, which impact SARS-CoV-2 disease severity [7–17]. Viral factors are also important, especially as variants emerge with distinctive properties affecting pathogenesis and immune evasion. For example, the Delta variant was associated with high risk of ICU admission and mortality [11,18,19], while Omicron showed low neutralization sensitivity to vaccine induced immunity [20].

In order to elucidate viral and host factors that contribute to disease outcomes in the context of low vaccination rates, we analyzed demographic and clinical data from 1,957 individuals with SARS-CoV-2 infection in the state of Georgia during the Delta and Omicron waves. We also analyzed full viral genome sequences from 1,185 of these individuals to examine the influence of variants and mutations on disease severity. We leveraged a large study population and extensive demographic, clinical, and sequence data to define factors associated with SARS-CoV-2 disease severity in a region marked by low vaccination rates.

## Results

### Clinical and demographic factors differ by SARS-CoV-2 vaccine status

**Fig 1:**
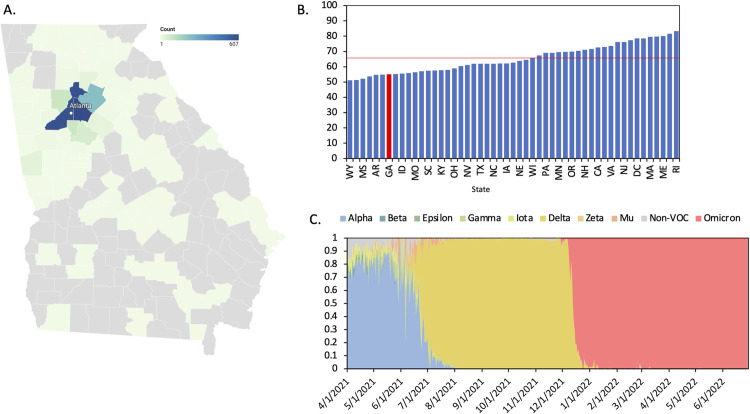
COVID-19 cases from May 2021-May 2022 in Georgia, US. **A.** 1,957 Emory Healthcare COVID-19 case mapped by counties in and around Atlanta, GA. Created using Datawrapper **B**. Percent of population fully vaccinated against SARS-CoV-2 by May 2022 in each state in the United States. Red line represents national average (66.7%). Red bar represents Georgia. **C.** Proportion of variants circulating in Georgia from April 2021- June 2022. Sequences obtained from GISAID.

Between May 2021 and May 2022, we identified 1,957 individuals who tested positive for SARS-CoV-2 within the Emory Healthcare system in Atlanta, Georgia, during a time when universal screening was performed for all hospitalizations and Emergency Department (ED) visits, regardless of symptoms. We performed detailed manual review of the Electronic Medical Records (EMR) for each individual. The majority of individuals (66%) were residents of the Metro Atlanta area (Table 1, Fig 1A). The median age was 51 years (Interquartile range [IQR] = 36,65), and 56% of individuals were female. The racial distribution of participants was predominantly Black (58%) or White (31%). Individuals experienced a range of clinical presentations and outcomes, from asymptomatic infection (15%) to death (2.8%) (Table 2). Among the 967 individuals in this study who were hospitalized, 625 (65%) of the hospitalizations were due to COVID-19.

**Table 1:** Demographic characteristics by vaccination status.

Variable	Overall, N = 1,957^1^^,^^2^	Unvaccinated, N = 1,024^1^^,^^2^	Vaccinated, N = 933^1^^,^^2^	p^3^^,^^4^
**Age**				
Age (in years)	51 (36, 65)	43 (31, 59)	58 (43, 70)	<0.001
Age (by decade):				<0.001
18-29	275 (14%)	216 (21%)	59 (6.3%)	
30-39	342 (17%)	225 (22%)	117 (13%)	
40-49	325 (17%)	178 (17%)	147 (16%)	
50-59	317 (16%)	152 (15%)	165 (18%)	
60-69	332 (17%)	133 (13%)	199 (21%)	
70-79	228 (12%)	80 (7.8%)	148 (16%)	
80-89	113 (5.8%)	31 (3.0%)	82 (8.8%)	
90 +	25 (1.3%)	9 (0.9%)	16 (1.7%)	
**Sex**				0.10
Female	1,093 (56%)	590 (58%)	503 (54%)	
Male	864 (44%)	434 (42%)	430 (46%)	
**Race**				<0.001
American Indian/Alaska Native	64 (3.3%)	23 (2.2%)	41 (4.4%)	
Asian/Native Hawaiian/ Pacific Islander	3 (0.2%)	1 (<0.1%)	2 (0.2%)	
Black	1,137 (58%)	721 (70%)	416 (45%)	
White	611 (31%)	211 (21%)	400 (43%)	
Other	6 (0.4%)	5 (0.5%)	1 (0.1%)	
Unknown	134 (6.9%)	62 (6.1%)	72 (7.7%)	
(Missing)	2	1	1	
**Ethnicity**				0.99
Hispanic/Latino	64 (3.6%)	34 (3.6%)	30 (3.6%)	
Non-Hispanic/Latino	1,726 (96%)	918 (96%)	808 (96%)	
(Missing)	167	72	95	
**Residence Region**				0.28
Metro Atlanta	1,301 (66%)	692 (68%)	609 (65%)	
Other	656 (34%)	332 (32%)	324 (35%)	

^1^N = number of participants

^2^N (%) or median (Interquartile Range [IQR])

^3^p = p-value for staticial significance

^4^Pearson’s Chi-squared test or Fisher’s exact test for categorical variables and Wilcoxon rank-sum test for continuous variables

**Table 2:** Clinical characteristics by vaccination status.

Variable	Overall, N = 1,957^1^^,^^2^	Unvaccinated, N = 1,024^1^^,^^2^	Vaccinated, N = 933^1^^,^^2^	p^3^^,^^4^
**COVID Symptoms**				
Any Systemic Symptoms^5^	1,301 (82%)	713 (84%)	588 (81%)	0.08
(Missing)	379	175	204	
Any GI Symptoms^6^	642 (42%)	387 (47%)	255 (37%)	<0.001
(Missing)	440	205	235	
Any Upper Respiratory^7^ Symptoms	636 (43%)	281 (35%)	355 (53%)	<0.001
(Missing)	474	216	258	
Any Lower Respiratory^8^ Symptoms	1,275 (80%)	675 (80%)	600 (80%)	0.71
(Missing)	364	177	187	
**Symptom Duration at Time of Testing**				<0.01
0-3 days	681 (43%)	338 (39%)	343 (48%)	
4-7 days	428 (27%)	255 (29%)	173 (24%)	
8 + days	238 (15%)	147 (17%)	91 (13%)	
Asymptomatic	233 (15%)	125 (14%)	108 (15%)	
(Missing)	377	159	218	
**Underlying Conditions**				
Any Immunosuppressed Underlying Condition^9^	407 (22%)	162 (17%)	245 (28%)	<0.001
(Missing)	145	73	72	
Any Other Underlying Condition^10^	1,164 (65%)	573 (60%)	591 (70%)	<0.001
(Missing)	155	71	84	
**Disease Severity**				0.70
Mild	1,148 (62%)	609 (62%)	539 (62%)	
Moderate	463 (25%)	251 (26%)	212 (24%)	
Severe	182 (9.9%)	92 (9.4%)	90 (10%)	
Death	51 (2.8%)	24 (2.5%)	27 (3.1%)	
(Missing)	113	48	65	
**Vaccination**				
Days Since Full Vaccination	198 (141, 272)	—	198 (141, 272)	
(Not Applicable)	1,024	1,024	—	
Days Since Booster	115 (63, 163)	—	115 (63, 163)	
(Not Applicable)	1,796	1,024	772	
Days Since Most Recent Vaccination/Booster				
Unvaccinated	1,024 (52%)	1,024 (100%)	—	
Within past 90 Days	136 (6.9%)	—	136 (15%)	
91-180 Days Ago	402 (21%)	—	402 (43%)	
181-270 Days Ago	283 (14%)	—	283 (30%)	
More than 270 Days Ago	112 (5.7%)	—	112 (12%)	

^1^N = number of participants

^2^N (%) or median (Interquartile Range [IQR])

^3^p = p-value for staticial significance

^4^Pearson’s Chi-squared test or Fisher’s exact test for categorical variables and Wilcoxon rank-sum test for continuous variables

^5^Any Systemic symptoms included fatigue, fever, chills, rigors, myalgia, and headache

^6^Any GI symptoms included nausea/vomiting, and diarrhea

^7^Any Upper Respiratory symptoms included sore throat, runny nose, and nasal congestion

^8^Any Lower Respiratory symptoms included cough, shortness of breath, and difficulty breathing

^9^Any Immunosuppressed Underlying Condition included HIV, active cancer, autoimmune disease, immunosuppressed, or immunosuppressive therapy

^10^Any Other Underlying Condition included overweight, diabetes, renal, cardiovascular, pregnant and liver disease

During the period of this study, the state of Georgia had the 7^th^ lowest vaccination rate in all 50 United States and the District of Columbia, with 55.1% of the population vaccinated (Fig 1B). In our study, a slightly lower proportion (48%) of individuals were fully vaccinated, in part because our study was designed to ensure inclusion of unvaccinated individuals. Booster doses became available to all adults in November 2021, however, of the fully vaccinated individuals in this study who were infected after the booster was available (n = 396), only 38% (n = 152) had received the booster and were considered “up-to-date” on vaccinations. Vaccinated individuals were significantly older than unvaccinated individuals (median age of 58 years vs 43 years, p < 0.001) (Table 1). We observed a large disparity in vaccine status by race; among the unvaccinated participants, 70% were Black and 21% White, while among the vaccinated participants, 45% were Black and 43% were White (Table 1). Vaccination status was not significantly associated with any other demographic variable evaluated.

We found that vaccinated individuals were more likely to have underlying medical comorbidities than unvaccinated individuals. Notably, 28% of vaccinated individuals were immunocompromised, compared to 17% of unvaccinated individuals (p < 0.001, Table 2). Vaccinated individuals were also more likely to have hypertension (56% vs. 39%, p < 0.001), cardiovascular disease (36% vs. 23%, p < 0.001), diabetes (28% vs. 19%, p < 0.001), renal disease (24% vs. 11%, p < 0.001), and autoimmune disease (7% vs. 4%, p =  0.03) (S1 Table). Vaccinated individuals were less likely to be pregnant (1% vs. 6%, p < 0.001) (Table 2, S1 Table), however, at the time of the study, vaccines were not yet approved for pregnant individuals. These findings are consistent with higher rates of vaccination in individuals with medical comorbidities.

We also found differences in clinical symptoms by vaccination status. Compared to unvaccinated individuals, those who were vaccinated were less likely to have fever (52% vs. 42%, p < 0.001), chills (38% vs. 32%, p =  0.02), nausea/vomiting (35% vs. 25%, p < 0.001), and shortness of breath or difficulty breathing (49% vs. 39%, p < 0.001); they were more likely to have sore throat (17% vs. 22%, p =  0.02) and runny nose/nasal congestion (27% vs. 45%, p < 0.001) (S1 Table). These findings are consistent with milder disease in vaccinated individuals.

### Variant frequency differs between vaccinated and unvaccinated individuals

We sequenced full SARS-CoV-2 genomes from residual nasopharyngeal swab samples from 1,185 individuals. The minimum genome coverage of the samples was 76% and the median sequencing depth was 1,804 (S1 Dataset). Sequences were primarily Delta (68%) and the BA.1 sublineage of the Omicron variant (23%), followed by other Omicron sublineages (5.1%), Alpha (2.5%) and less than 1% each of Beta, Gamma, Mu, A.2.5, and B.1 (Table 3). In Georgia, during the time of this study (May 2021- May 2022), Delta accounted for 55% of infections, Omicron for 40%, and Alpha for 2.5% (Fig 1C, S2 Table). Therefore, our study included a somewhat higher proportion of Delta and a lower proportion of Omicron than was circulating in the state. The distribution of all other variants aligned with the overall variant distribution observed in Georgia (S1 Table, Table 3).

**Table 3:** SARS-CoV-2 variants by vaccination status.

Variable	Overall, N = 1,957^1^^,^^2^	Unvaccinated, N = 1,024^1^^,^^2^	Vaccinated, N = 933^1^^,^^2^	p ^3^^,^^4^
**Lineage**				<0.001
A.2.5	1 (<0.1%)	0 (0%)	1 (0.2%)	
Alpha	30 (2.5%)	24 (4.0%)	6 (1.0%)	
B.1	1 (<0.1%)	0 (0%)	1 (0.2%)	
Beta	1 (<0.1%)	0 (0%)	1 (0.2%)	
Delta	805 (68%)	415 (70%)	390 (66%)	
Gamma	6 (0.5%)	5 (0.8%)	1 (0.2%)	
Mu	4 (0.3%)	4 (0.7%)	0 (0%)	
Omicron	337 (28%)	146 (25%)	191 (32%)	
(Missing)	772	430	342	

^1^N = number of participants

^2^N (%) or median (Interquartile Range [IQR])

^3^p = p-value for staticial significance

^4^Fisher’s exact test

The distribution of SARS-CoV-2 variants in our study was different for vaccinated and unvaccinated individuals (p < 0.001) (Table 3). Omicron had a higher frequency in vaccinated individuals (32%) than unvaccinated (25%), whereas Alpha and Delta were more common in unvaccinated compared to vaccinated individuals (Table 3). These differences correspond with the timing of each variant’s circulation compared to vaccine rollout, though decreased vaccine effectiveness against Omicron may also contribute [21].

**Fig 2:**
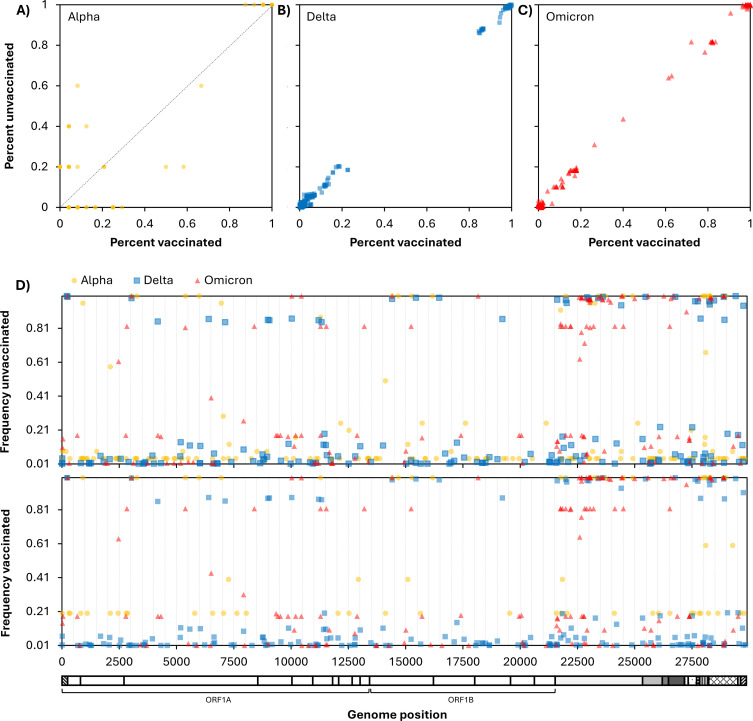
Frequencies of single nucleotide polymorphisms (SNPs) among SARS-COV-2 genome sequences from vaccinated and unvaccinated individuals. Each point represents a single SNP plotted by its frequency in sequences from unvaccinated individuals (Y-axis) versus its frequency in sequences from vaccinated individuals (X-axis). Data is divided by WHO variant classifications Alpha (A), Delta (B), and Omicron (C). Mutations observed along the diagonal depict mutations observed equally among vaccinated and unvaccinated individuals. Mutations observed moving away from the diagonal represent mutations observed in either vaccinated (X-axis) or unvaccinated (Y-axis) individuals. (D) Frequency of SNPs in SARS-CoV-2 sequences from unvaccinated (top) and vaccinated (bottom) individuals, by genome position (x-axis). In all panels, Alpha is represented by yellow circles, Delta by blue squares, and Omicron by red triangles.

In addition to the frequency of VOCs among vaccinated and unvaccinated individuals, we investigated the frequency of individual mutations. Within each VOC – Alpha, Delta, and Omicron – no sequence characteristics, including mutations, deletions, and insertions, were different between viruses infecting vaccinated and unvaccinated individuals (Fig 2, S1 Fig). However, the number of non-lineage-defining mutations was lower in vaccinated compared to unvaccinated individuals (Fig 1D), suggesting that vaccination may have an impact on viral diversity within a host. Phylogenetic analysis demonstrated that sequences from vaccinated individuals were intermixed with sequences from unvaccinated individuals, further confirming no distinct features of post vaccination infections (S2 Fig).

Metagenomic analysis of RNA sequence data from 513 samples identified one sample with rhinovirus A, one with coxsackievirus A21, and one with adenovirus (S1 Dataset). As this was a convenience analysis that did not include EMR review, we have likely missed co-infections especially with bacteria and fungi. Nevertheless, our results are consistent with other studies that have shown low rates of viral co-infection with SARS-CoV-2 [22,23].

### Age, underlying conditions, and vaccination status are associated with disease severity

We evaluated associations between disease severity and demographic characteristics, underlying health conditions, vaccination status, and SARS-CoV-2 variant. Disease severity was defined according to the WHO clinical progression scale using data obtained by manual chart review [24]. Mild disease included asymptomatic infection and symptomatic infection without hospitalization. Moderate disease included hospitalized individuals without oxygen therapy or oxygen by mask or nasal prongs. Severe disease included the use of oxygen by noninvasive or high flow, intubation and mechanical ventilation, vasopressors, dialysis, or extracorporeal membrane oxygenation. Death included in-hospital deaths directly linked to COVID based on manual chart review.

Using an adjusted multinomial logistic regression model that included demographic characteristics, underlying conditions, vaccination status and variant, we found that age, certain underlying medical conditions, and time since most recent vaccination were significantly associated with disease severity (Table 4). Underlying health conditions significantly associated with disease severity in the adjusted model included chronic lung disease, renal disease, and the use of systemic immunosuppressive therapy prior to hospitalization. Other underlying health conditions were associated with SARS-CoV-2 disease severity in unadjusted univariate analyses, including hypertension, overweight, cardiovascular disease, diabetes, liver disease, and autoimmune disease (S3 Table), but these were no longer significant after adjustment for other factors.

**Table 4:** Adjusted multinomial logistic regression models testing the association between demographics, underlying health conditions, and COVID-related characteristics with disease severity.

	Moderate^1^			Severe^1^			Death^1^		
**Variable**	**aOR** ^2^	**95% CI** ^2^	**p** ^3^ ^,^ ^4^	**aOR** ^2^	**95% CI** ^2^	**p** ^3^ ^,^ ^4^	**aOR** ^2^	**95% CI** ^2^	**p** ^3^ ^,^ ^4^
**Demographics**									
***Age***	***1.05***	***1.03, 1.06***	***<0.001***	***1.05***	***1.03, 1.07***	***<0.001***	***1.09***	***1.05, 1.13***	***<0.001***
Male	1.28	0.89, 1.85	0.18	1.34	0.82, 2.19	0.24	1.21	0.52, 2.81	0.66
White	0.70	0.46, 1.06	0.09	0.89	0.51, 1.54	0.67	0.73	0.27, 1.96	0.53
**Underlying Conditions**									
***Chronic Lung Disease***	***2.10***	***1.38, 3.17***	***<0.001***	***1.98***	***1.15, 3.41***	***0.01***	1.31	0.50, 3.46	0.58
Hypertension	1.02	0.65, 1.59	0.94	0.97	0.53, 1.76	0.91	3.46	0.88, 13.57	0.08
Overweight	1.40	0.95, 2.07	0.09	1.17	0.69, 1.99	0.56	1.61	0.65, 4.01	0.30
***Cardiovascular Disease***	***1.63***	***1.06, 2.50***	***0.03***	1.45	0.82, 2.55	0.20	1.08	0.44, 2.63	0.87
Diabetes	0.99	0.64, 1.54	0.98	1.45	0.83, 2.54	0.20	1.09	0.43, 2.74	0.86
***Renal Disease***	***2.64***	***1.61, 4.32***	***<0.001***	***1.91***	***1.01, 3.64***	***0.05***	***3.63***	***1.41, 9.36***	***0.01***
Liver Disease	1.13	0.48, 2.69	0.78	1.84	0.69, 4.92	0.22	1.48	0.25, 8.78	0.67
Autoimmune Disease	1.78	0.87, 3.65	0.11	1.95	0.77, 4.95	0.16	3.96	1.00, 15.65	0.05
Immunocompromised^5^	1.08	0.55, 2.11	0.82	0.59	0.25, 1.40	0.23	3.26	0.83, 12.71	0.09
***Systemic Immunosuppressive Therapy or Meds***	***3.45***	***1.88, 6.32***	***<0.001***	***5.01***	***2.38, 10.51***	***<0.001***	1.48	0.38, 5.71	0.57
**Days Since Most Recent Vaccination/Booster**									
Unvaccinated	Ref	—		Ref	—		Ref	—	
***Within past 90 Day*** ^6^	***0.37***	***0.16, 0.88***	***0.02***	0.40	0.13, 1.26	0.12	0.72	0.18, 2.86	0.64
***91-180 Days Ago*** ^6^	***0.32***	***0.19, 0.54***	***<0.001***	***0.42***	***0.21, 0.82***	***0.01***	***0.31***	***0.10, 0.96***	***0.04***
***181-270 Days Ago*** ^6^	***0.32***	***0.18, 0.56***	***<0.001***	***0.38***	***0.17, 0.80***	***0.01***	***0.14***	***0.04, 0.59***	***0.01***
More than 270 Days^6^Ago	0.53	0.23, 1.21	0.13	0.88	0.34, 2.29	0.79	0.34	0.06, 1.95	0.23
**Lineage**									
Delta	Ref	—		Ref	—		Ref	—	
Omicron^7^	0.95	0.61, 1.47	0.81	1.42	0.81, 2.50	0.22	2.07	0.84, 5.12	0.12

1” Mild” is the Reference Category

^2^aOR =  Adjusted Odds Ratio, CI =  Confidence Interval, Ref =  Reference Level

^3^p = p-value for staticial significance

^4^Pearson’s Chi-squared test or Fisher’s exact test for categorical variables and Wilcoxon rank-sum test for continuous variables

^5^HIV infection, active cancer, solid organ transplant, hematopoietic stem cell transplant

^6^Compared to unvaccinated individuals

^7^Compared to Delta

Vaccination was protective in this population. In the adjusted model, vaccination within 91-180 days (about 3 – 6 months) was associated with lower odds of moderate disease (OR = 0.32, 95% CI 0.19-0.54), severe disease (OR = 0.42, 95% CI 0.21-0.82), and death (OR = 0.31, 95% CI 0.10-0.96) compared to unvaccinated individuals (Table 4). There were similar effects across all disease severity outcomes when vaccination occurred between 181 – 270 days (about 6 to 9 months). However, when vaccination occurred more than 270 days prior to infection, there were no significant differences in disease severity when compared to the unvaccinated group. Additionally, vaccination that had occurred within the past 90 days (about 3 months) was associated with lower odds of moderate disease relative to mild disease, but we did not find evidence of an association between vaccination and severe disease or death at that timepoint. In short, vaccinations were most protective against moderate disease, severe disease,

and death when they occurred within the prior 3-9 months.

The Omicron variant did not show a significant association with disease severity compared to the Delta variant in the adjusted model (Table 4). In the unadjusted model, Omicron was associated with higher disease severity than Delta (S3 Table), however we believe this could be due to the fact that individuals infected with Omicron had a longer interval between vaccination and infection. A post hoc analysis of the data showed that the average number of days since vaccination among those with Omicron was greater than among those with Delta lineage (median = 208 vs 170, Wilcoxon rank sum p < 0.001).

Overall, after controlling for multiple host and viral factors, we found that age, chronic lung disease, renal disease, and immunosuppressive therapy increased the odds of progressively more severe COVID-19 disease, while vaccination decreased the odds, and SARS-CoV-2 variant had no effect.

## Discussion

Our comprehensive analysis of host and viral factors associated with SARS-CoV-2 disease severity in a setting with low vaccination rates led to several key findings. First, age and underlying health conditions – especially chronic lung disease, renal disease, and the use of immunosuppressive therapy – were associated with more severe disease and death. Second, SARS-CoV-2 variant and viral mutations were not associated with disease severity in this study population, which was comprised of individuals who sought any medical care at Emory Healthcare hospitals. And third, vaccination was protective against severe outcomes for both Delta and Omicron variants. Unique features of our study included the analysis of a large number of SARS-CoV-2 full viral genome sequences linked to extensive clinical and demographic data, and our focus on a relatively under-studied region of the U.S.

Georgia is an important proxy for the southeastern U.S. and other populations with high numbers of vaccine refusals, inequitable access to healthcare, and low insurance coverage [25–27]. Emphasizing the positive impact of SARS-CoV-2 vaccination among this population, similar to others in the U.S. [28,29], is critical as new variants emerge. It is also important to note that among the individuals in this study who contracted SARS-CoV-2 after being vaccinated, a greater proportion reported milder upper respiratory symptoms such as sore throat and runny nose, while a lower percentage experienced more severe symptoms such as nausea/vomiting, fever, and shortness of breath/difficulty breathing, in comparison to unvaccinated individuals. Vaccinations were most protective against severe COVID outcomes when they occurred within the prior 3-9 months. This finding has timely implications on a national level, given persistently low vaccine uptake, especially of the bivalent SARS-CoV-2 vaccine [5], and the need for ongoing vaccine updates targeting emerging variants [4]. Indeed, we found that 62% of vaccinated individuals infected in or after November 2021 were not “up-to-date” on SARS-CoV-2 vaccinations. Multiple studies have found that SARS-CoV-2 vaccines wane after about 6 months [30], however, booster doses extend the duration of vaccine induced immunity [31], further emphazing the value of booster doses. Results from our study will help emphasize the benefits of vaccination to the public as a means of safeguarding against severe COVID outcomes.

Our results also indicated the importance of demographic and clinical factors associated with SARS-CoV-2 disease severity, despite vaccination. Age was a key risk factor; after accounting for vaccination status, demographic factors, health conditions, and SARS-CoV-2 variant, our analysis revealed that for each additional year of age, the odds of experiencing more severe outcomes compared to mild disease increased by 5%. The association between age and disease severity has been consistently observed, particularly among individuals aged 65 and above [7–10]. Thus, relying solely on vaccination may be insufficient for reducing disease severity and mortality among older individuals. U.S. Census Bureau data indicates that the population is aging, with Georgians aging at an even faster rate [32], underscoring the need for additional preventative and treatment measures.

In addition to age, we found that chronic lung disease, renal disease, and the use of immunosuppressive therapy also increased the odds of experiencing moderate and/or severe infection and/or death. In previous studies, cardiovascular disease, diabetes, chronic respiratory conditions, obesity, and compromised immune systems have also been found to increase the risk of severe illness [10–17].

It is surprising that we found no difference in disease severity between Delta and Omicron variants, since multiple prior studies have found that the risk of hospitalization, ICU admission, and mortality vary by variant [11,18,19,33]. This discrepancy may be due to the fact that all individuals in our study sought medical care, so we did not include individuals with minimal symptoms, which is a limitation of our study design. Similar to our results, another study of hospitalized patients found that vaccination was similarly protective for individuals with both Delta and Omicron variants [34]. Additionally, in this study, there was no evidence of an association between vaccination within 90 days of infection and the most severe outcomes (severe disease and death), though an association has been found in previous studies [12,17,30]. This finding could be due to immunity from natural infection in the unvaccinated group. Indeed, a recent meta-analysis showed that protection against severe disease from past infection remained high even at 40 weeks in unvaccinated individuals [35]. Due to the study design, we could not collect reliable data regarding prior SARS-CoV-2 infection(s) thus, we were unable to account for natural immunity.

We did not find viral factors associated with post-vaccine infection. Most post-vaccine infections were caused by the predominant lineage of the time. Within each variant (Delta and Omicron), no SARS-CoV-2 SNPs, deletions, or insertions were more common in vaccinated individuals than unvaccinated individuals. These results are different from a prior study of similar size, which found more resistance mutations (e.g., L452 * and E484*) in vaccinated compared to unvaccinated individuals in the pre-Omicron era [16]. Our negative finding likely reflects the challenge of identifying the effect of an individual virus mutation in an increasingly complex immune landscape. Interestingly, we found that vaccinated individuals had fewer non-lineage-defining SNPs than unvaccinated individuals, suggesting less diversity and potentially less viral evolution within vaccinated individuals. This is consistent with a recent study investigating within-host genetic diversity of SARS-CoV-2 in unvaccinated and vaccinated individuals [36]. It will be crucial to continue to monitor associations between viral genomic features and disease severity as new variants and mutations continue to arise.

Our study had several limitations: We only included individuals who presented to care, thus skewing our study population towards individuals with more severe disease than the general population. Our study population included few individuals in the following demographic categories: Asian/Native Hawaiian/Pacific Islander, and Hispanic/Latino. Therefore, our sample is not representative of the general population in terms of clinical and demographic factors. Given the retrospective study design, there may be residual confounding, however we adjusted for important variables such age, demographics, pre-existing health conditions, variant, and vaccination status. Lastly, do to the timing of our study, our results are biased towards Delta and early Omicron variants. While we were able to capture the initial roll out of booster doses, our study period did not include the introduction of the bivalent vaccine against the Omicron variants.

In summary, our findings underscore the critical role of vaccination status, age, and medical comorbidities – especially immunosuppression, chronic kidney disease, and chronic lung disease – in determining disease severity and outcomes among SARS-CoV-2 infected individuals, regardless of virus variant. We contribute valuable insights into the nuanced relationship between these factors, highlighting the importance of considering demographic, clinical, and genetic variables when evaluating disease severity. Ultimately, our results will be valuable in strengthening and reinforcing messaging around SARS-CoV-2 vaccination, especially in settings of low vaccine uptake.

## Materials and methods

### Clinical and demographic data

This study was approved by the institutional review board at Emory University under protocol STUDY00000260, with a waiver of consent. Emory Healthcare system is comprised of a network of hospitals and clinics throughout the metro Atlanta area and surrounding counties. During the duration of sample and data collection, Emory Healthcare required universal SARS-CoV-2 testing, including for emergency department visits, appointments, admissions, and surgeries. All positive SARS-CoV-2 samples that were tested at the Emory University Hospital Molecular and Microbiology Laboratories collected between May 3, 2021 and May 31, 2022 were reviewed for inclusion in this study.

The Centers for Disease Control and Prevention (CDC)-funded Georgia Emerging Infections Program (GA EIP) performs active, population- and laboratory- based surveillance for hospitalized cases of SARS-CoV-2 in metropolitan Atlanta, GA (population ~ 4 million). Patient vaccination status was retrieved by GA EIP from the Georgia Registry of Immunization Transactions and Services (GRITS) database. Individuals were considered vaccinated if they had received a complete vaccine series (2 doses of the Pfizer-BioNTech or Moderna vaccines or 1 dose of the Janssen vaccine) at least 14 days before their first positive test result and “Up-to-date” if they received at least 1 booster dose of the Pfizer-BioNTech or Moderna vaccines. State of residency was retrieved from Georgia’s State Electronic Notifiable Disease Surveillance System (SENDSS). Individuals were excluded from the study if they were partially vaccinated or reported out-of-state residency. From May 2021- September 2021, individuals were included in the study on a case-match basis; for each post-vaccine case identified, 2-3 non-vaccinated control cases were selected at random from the positive samples tested in the same calendar week. From October 2021- May 2022, all SARS-CoV-2 positive individuals identified who met inclusion criteria were included. Patient demographics, underlying medical conditions, symptomatic illness, hospitalization, and disease outcome were obtained from manual review of the electronic medical record (EMR). The electronic medical records (EMRs) of patients with hospitalization and/or death were carefully reviewed to determine whether these outcomes were directly attributable to COVID-19. In constructing symptom categories, systemic symptoms were defined as fatigue, fever, chills, rigors, myalgia, and headache. Gastrointestinal symptoms were defined as nausea/vomiting and diarrhea. Upper respiratory symptoms were defined as sore throat, runny nose, and nasal congestion. Lower respiratory symptoms were defined as cough, and shortness of breath/difficulty breathing. Immunosuppressed was defined as HIV, active cancer, autoimmune disease, or immunosuppressive therapy. Systemic immunosuppressive therapy included chemotherapy, corticosteroids, and anti-CD20 monoclonal antibodies. During chart review, all underlyng medical conditions were reported as binary variables (yes or no), and the specific type and duration of therapy was not recorded for each individual. “Other” pre-existing conditions were defined as overweight, diabetes, renal, cardiovascular, pregnant, and liver disease. When specific information was not available in the EMR for an individual, this information was recorded as “missing”, and the number of individuals with missing information for each variable is presented in our tables. Variables with no missing data include age, vaccination status, sex, and GA residency.

Disease severity was defined according to the WHO clinical progression scale [24]. The scale includes mild disease- asymptomatic and symptomatic SARS-CoV-2 infection without hospitalization, moderate disease- hospitalization without oxygen therapy or hospitalization with oxygen by mask or nasal prongs, severe disease- hospitalization with use of oxygen by noninvasive or high flow, intubation and mechanical ventilation, vasopressors, dialysis, or extracorporeal membrane oxygenation, and death. The WHO clinical progression scale disease severity for each individual was determined based on data collected by manual chart review.

Data management and cleaning were conducted in Excel v16.73 and SAS studio v3.81.

### SARS-CoV-2 sequencing and analysis

Residual nasopharyngeal (NP) swab samples were obtained from the Emory University Hospital Molecular and Microbiology Laboratories. NP samples with a Ct of 32 or lower underwent RNA extraction, DNase treatment, and cDNA synthesis followed by metagenomic or amplicon-based library construction. For metagenomic sequencing, Nextera XT (Illumina) and Illumina sequencing were performed as previously described [37]. Amplicon-based sequencing was performed using the xGEN SARS-CoV-2 kit (IDT) as previously described [38].

Reference-based SARS-CoV-2 genome assembly was performed using viral-ngs v2.1.12.0 [39] or Viralrecon [40] for metagenomic and amplicon sequencing, respectively, with reference strain NC_045512. SARS-CoV-2 lineages were determined using Pangolin [41]. Sequences were aligned and visualized in Geneious Prime (https://www.geneious.com). Consensus-level single nucleotide polymorphisms (SNPs) and insertions/deletions were identified using the Nextstrain web-based mutation calling tool [42]. Sequences were included in analyses if they had at least 75% genome coverage and at least 10X depth, and most of the included sequences exceeded these thresholds; 1166 (98%) of samples had more than 95% genome coverage, and 77% of samples had more than 100X depth. Coverage and depth for each sample is listed in the S1 Dataset.

For phylogenetic analysis, 411,634 reference sequences, collected between May 1, 2021, and May 31, 2022, were downloaded from NCBI and were aligned with our study sequences to reference strains Wuhan/Hu-1/2019 and Wuhan/WHO/2019 using Nextalign within the Nextstrain v3.2.4 pipeline 7. This dataset was subsampled in Nextstrain using a custom scheme, in which crowd penalty was set to 0.0 to select 1000 sequences most genetically similar to our sequence dataset. Maximum likelihood phylogenetic trees were constructed using default settings of the Nextstrain SARS-CoV-2 Workflow with TreeTime v0.8.6 [43].

### Viral metagenomic analysis

To assess the presence of other respiratory viruses in 513 samples that underwent metagenomic sequencing, reads were first passed through a pre-processing pipeline including deduplication with Clumpify.sh in the BBMap tools (https://sourceforge.net/projects/bbmap/). Deduplicated reads were trimmed with Trimmomatic Version 0.40 and filtered for quality, with flags leading:3, trailing:3, slidingwindow:4:15, minlen:36 (https://github.com/usadellab/Trimmomatic). Pre-processed reads were run through kraken2 v2.1.3 against the k2_pluspf_20210127 database to assign each read to a taxonomic group, then adjusted for significance with Bracken [44,45]. Within the Kraken Tools packages, the extract_kraken_reads.py script was used to separate reads by taxonomic ID for human taxID_hg = ”9606”, bacteria taxID_bac = ”2”, fungus taxID_fungus = ”4751”, viruses taxID_virus = ”10239”, and COVID-19 taxID_COVID = ”2697049”. Custom shell and R scripts were used to determine if the following viruses were found in each sample:

Human mastadenovirus C taxID = 129951, Coronavirus HKU1 taxID = 443239, Coronavirus NL63 taxID = 277944, Coronavirus 299E taxID = 11137, Coronavirus OC43 taxID = 31631, SARS-CoV-2 taxID = 2697049, Paramyxoviridae taxID = 11158, Human metapneumovirus taxID = 162145, Parainfluenza virus taxID = 2905673, Respiratory syncytial virus taxID = 12814, Picornaviridae taxID = 12058, Rhinovirus taxID = 31708, Enterovirus taxID = 12059, Orthomyxoviridae taxID = 11308, Influenza A taxID = 382835, and Influenza B taxID = 11520. Reads belonging to the above taxa were confirmed using BLASTN, the best-matching reference sequence on RefSeq was identified, and read mapping was performed in Geneious Prime (www.geneious.com). If reads mapped to at least 3 distinct genome regions, the virus was considered present in the sample.

### Statistical analysis

Demographics, symptoms, underlying conditions, and outcomes were described using frequency distributions for categorical variables and medians and interquartile ranges for continuous variables. Subgroup differences were evaluated to compare individuals who were vaccinated with individuals who were not vaccinated, using chi-square tests and Fisher’s exact tests for categorical variables and Wilcoxon rank-sum test for continuous variables.

Multinomial logistic regressions were used to test the association between demographic characteristics, underlying conditions, vaccine status, and SARS-CoV-2 variant with disease severity (1 = Mild [Reference Category], 2 = Moderate, 3 = Severe, 4 = Death). First, unadjusted models tested each variable’s association with disease severity separately. Next, multivariable models were constructed in a step-wise fashion, adding variables in blocks of demographic variables, including age (continuous), sex (0 = Female[Reference], 1 = Male), and race(0 = Black[Reference], 1 = White), followed by a block of underlying condition variables, all of which were binary (0 = No, 1 = Yes; pregnant, chronic lung disease, hypertension, overweight, cardiovascular disease, diabetes, renal disease, liver disease, autoimmune disease, immunocompromised, systemic immunosuppressive therapy/medications), and the final model added a block of SARS-CoV-2-related characteristics including vaccination status (1 = Unvaccinated [Reference], 2 = Vaccinated, 3 = Vaccinated and Boosted), Days Since Most Recent Vaccination/Booster (1 = Unvaccinated [Reference], 2 = Within past 90 Days, 3 = 91 – 180 Days Ago, 4 = 181 – 270 Days Ago, 5 = More than 270 Days Ago) and variant (1 = Delta[Reference], 2 = Omicron). The multivariable models were tested for multicollinearity using variance inflation factor (VIF), though collinearity was not present, so all variables were retained.

All analyses were conducted in R Version 4.1.3 (R Foundation for Statistical Computing, Vienna, Austria).

**Table:** Key resources table

REAGENT or RESOURCE	SOURCE	IDENTIFIER
**Biological samples **		
Residual NP swabs	Emory Microbiology Lab	N/A
**Critical commercial assays **		
Nextera XT DNA Library Preparation kit	Illumina	FC-131-1096
Illumina MiSeq kit	Illumina	MS-102-3001
Deposited data		
SARS-CoV-2 consequence sequences	This paper	GISAID: PRJNA634356
SARS-CoV-2 reads	This paper	NCBI: PRJNA634356
**Software and algorithms **		
Geneious		https://www.geneious.com
Nextstrain	Hadfield et al., 2018	https://clades.nextstrain.org/
IQtree	Trifinopoulos et al., 2016	http://iqtree.cibiv.univie.ac.at/
Interactive Tree of Life (iTOL)	Letunic et al., 2021	https://itol.embl.de/
ViReMA	Routh et al., 2013	https://sourceforge.net/projects/virema/
Pangolin	Rambaut et al., 2020	https://pangolin.cog-uk.io
Pilon	Walker et al., 2014	https://github.com/broadinstitute/pilon
Bowtie2	Langmead et al., 2012	http://bowtie-bio.sourceforge.net/index.shtml
samtools	Li et al., 2009	http://www.htslib.org/
seqtk	https://github.com/lh3/seqtk	https://github.com/lh3/seqtk
DESeq2	Love et al., 2014	https://bioconductor.org/packages/release/bioc/html/DESeq2.html
R Studios	RStudio: Integrated Development for R	*Rstudio.com*
SAS	SAS Institute	https://www.sas.com/en_us/home.html

## Supporting information

S1 Fig Mutations across SARS-CoV-2 genes. Alpha, delta, and omicron sequences were aligned to Wuhan/Hu-1/2019. For each variant, samples from vaccinated (“vax”) and unvaccinated (“unvax”) individuals are labeled. Each square in the heatmap represents the number of single nucleotide polymorphisms (SNPs) in each gene, labeled on the x-axis. Each row represents one sample.
(TIFF)

S2 Fig Phylogenetic analysis does not reveal differences in SARS-CoV-2 sequences from vaccinated and unvaccinated individuals. Maximum likelihood tree containing sequences from vaccinated (green) and unvaccinated (yellow) individuals in the context of 2000 global sequences from GISAID (orange) selected by a custom Nextstrain subsampling scheme and rooted to NC_045512.
(TIFF)

S1 Table Extended COVID symptoms and underlying conditions by vaccination status.
(DOCX)

S2 Table Circulation of Variants of Concern (VOC) and Variants of Interest (VOI) in Georgia, USA as measured by available sequences on GISAID.
(DOCX)

S3 Table Unadjusted multinomial logistic regression models testing the association between demographics, underlying health conditions, and COVID-related characteristics with disease severity.
(DOCX)

S1 Dataset SARS-CoV-2 genome sequencing metrics. (XLSX)
