# Supplementary figures and images for: Viral and host factors associated with SARS-CoV-2 disease severity in Georgia, USA
Source: PLoS One. 2025 Apr 1;20(4):e0317972. doi: 10.1371/journal.pone.0317972 (PMC11960886; doi:10.1371/journal.pone.0317972)

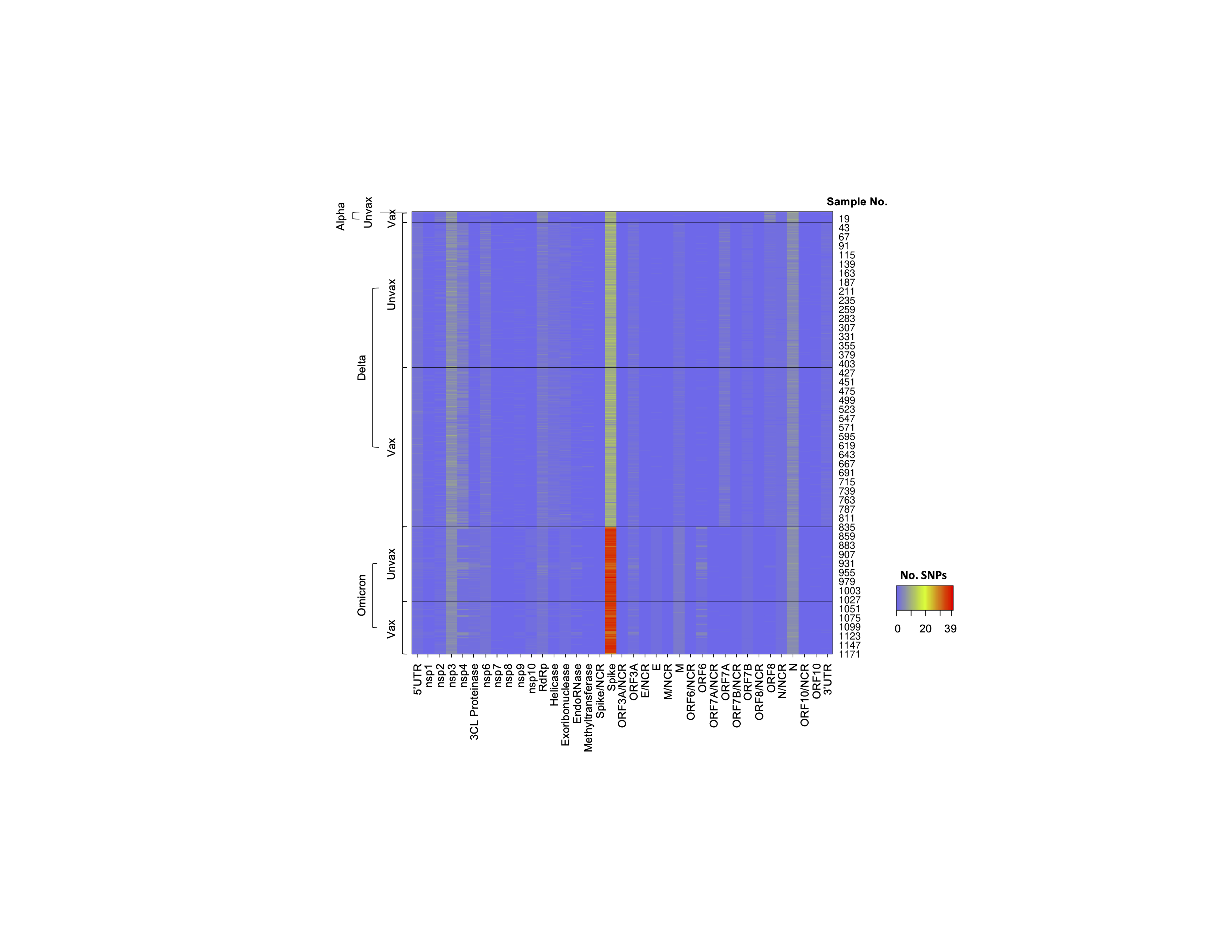

Supplement: S1 Fig — Alpha, delta, and omicron sequences were aligned to Wuhan/Hu-1/2019. For each variant, samples from vaccinated (“vax”) and unvaccinated (“unvax”) individuals are labeled. Each square in the heatmap represents the number of single nucleotide polymorphisms (SNPs) in each gene, labeled on the x-axis. Each row represents one sample. (TIFF) [file pone.0317972.s001.tiff]

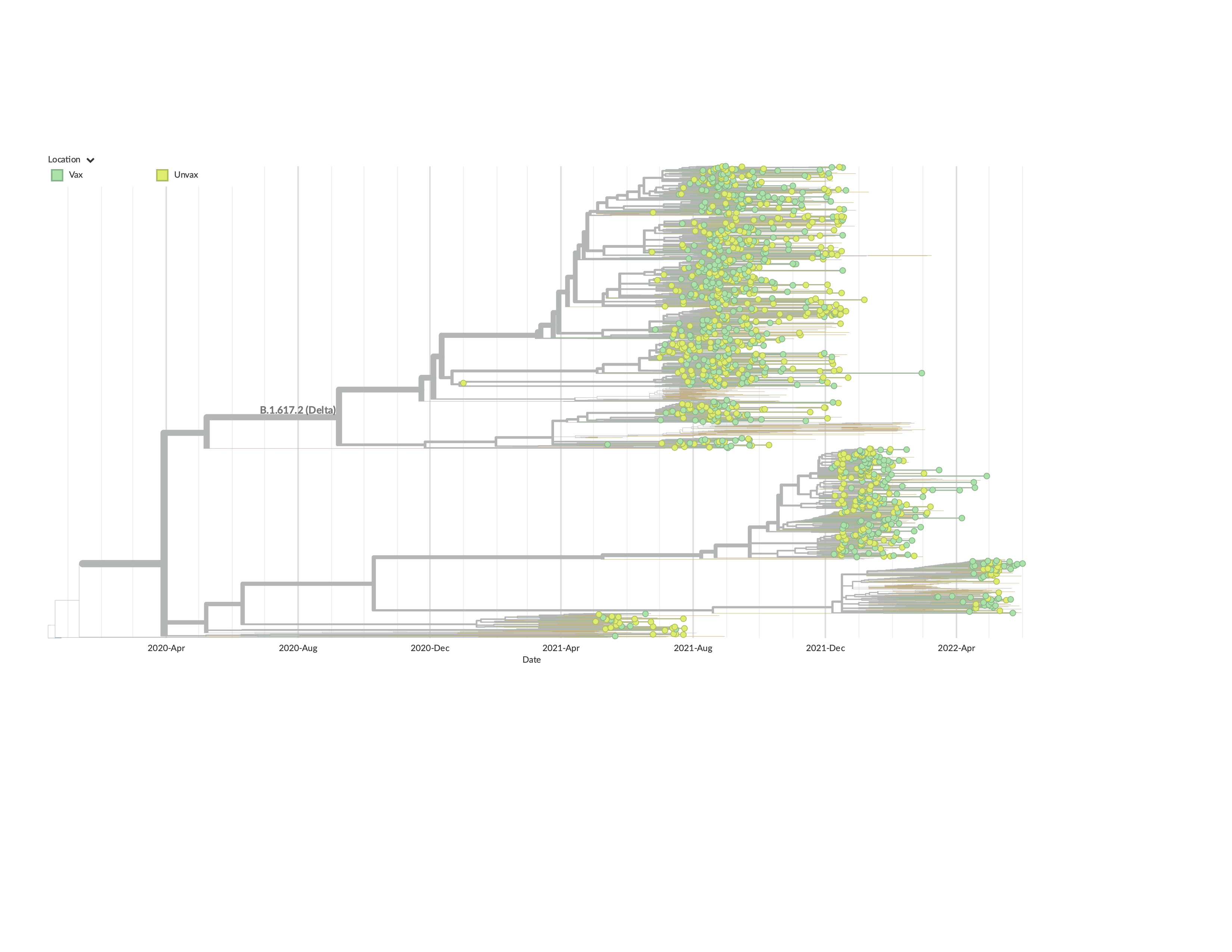

Supplement: S2 Fig — Maximum likelihood tree containing sequences from vaccinated (green) and unvaccinated (yellow) individuals in the context of 2000 global sequences from GISAID (orange) selected by a custom Nextstrain subsampling scheme and rooted to NC_045512. (TIFF) [file pone.0317972.s002.tiff]
