# Supplementary material for: Viral and host factors associated with SARS-CoV-2 disease severity in Georgia, USA
Source: PLoS One. 2025 Apr 1;20(4):e0317972. doi: 10.1371/journal.pone.0317972 (PMC11960886; doi:10.1371/journal.pone.0317972)
Supplement: S1 Table — (DOCX) [file pone.0317972.s003.docx]

| **Table S1. Extended COVID Symptoms and Underlying Conditions by Vaccination Status** | | | | |
| --- | --- | --- | --- | --- |
| **Variable** | **Overall**,  N = 1,957*^1,2^* | **Unvaccinated**,  N = 1,024*^1,2^* | **Vaccinated**,  N = 933*^1,2^* | **p** *^3,4^* |
| **COVID Symptoms** | | | | |
| Fever | 720 (47%) | 430 (52%) | 290 (42%) | <0.001 |
| (Missing) | 435 | 197 | 238 |  |
| Chills | 537 (35%) | 314 (38%) | 223 (32%) | 0.02 |
| (Missing) | 435 | 198 | 237 |  |
| Rigors | 29 (2.0%) | 17 (2.1%) | 12 (1.8%) | 0.67 |
| (Missing) | 491 | 221 | 270 |  |
| Myalgia | 501 (34%) | 279 (35%) | 222 (33%) | 0.59 |
| (Missing) | 484 | 218 | 266 |  |
| Headache | 521 (35%) | 299 (37%) | 222 (33%) | 0.12 |
| (Missing) | 476 | 215 | 261 |  |
| Sore Throat | 286 (20%) | 138 (17%) | 148 (22%) | 0.02 |
| (Missing) | 496 | 228 | 268 |  |
| Nausea / Vomiting | 451 (30%) | 282 (35%) | 169 (25%) | <0.001 |
| (Missing) | 455 | 209 | 246 |  |
| Diarrhea | 369 (25%) | 212 (26%) | 157 (23%) | 0.13 |
| (Missing) | 470 | 220 | 250 |  |
| Fatigue | 630 (41%) | 337 (41%) | 293 (41%) | 0.81 |
| (Missing) | 418 | 195 | 223 |  |
| Runny Nose or Nasal Congestion | 519 (35%) | 217 (27%) | 302 (45%) | <0.001 |
| (Missing) | 473 | 215 | 258 |  |
| Cough | 1,087 (70%) | 576 (69%) | 511 (72%) | 0.37 |
| (Missing) | 414 | 195 | 219 |  |
| Shortness of Breath or Difficulty Breathing | 690 (44%) | 408 (49%) | 282 (39%) | <0.001 |
| (Missing) | 401 | 188 | 213 |  |
| Loss of Taste | 264 (18%) | 151 (19%) | 113 (17%) | 0.33 |
| (Missing) | 505 | 233 | 272 |  |
| **Underlying Conditions** | | | | |
| Pregnant | 69 (3.6%) | 59 (6.0%) | 10 (1.1%) | <0.001 |
| (Missing) | 66 | 42 | 24 |  |
| Chronic Lung Disease | 387 (21%) | 193 (20%) | 194 (22%) | 0.33 |
| (Missing) | 134 | 75 | 59 |  |
| Hypertension | 874 (47%) | 377 (39%) | 497 (56%) | <0.001 |
| (Missing) | 95 | 50 | 45 |  |
| Overweight | 584 (32%) | 315 (33%) | 269 (32%) | 0.50 |
| (Missing) | 150 | 70 | 80 |  |
| Cardiovascular Disease | 534 (29%) | 217 (23%) | 317 (36%) | <0.001 |
| (Missing) | 126 | 65 | 61 |  |
| Diabetes | 426 (23%) | 180 (19%) | 246 (28%) | <0.001 |
| (Missing) | 101 | 53 | 48 |  |
| Renal Disease | 316 (17%) | 106 (11%) | 210 (24%) | <0.001 |
| (Missing) | 132 | 69 | 63 |  |
| Liver Disease | 83 (4.5%) | 37 (3.8%) | 46 (5.2%) | 0.16 |
| (Missing) | 107 | 59 | 48 |  |
| Autoimmune Disease | 101 (5.5%) | 42 (4.4%) | 59 (6.8%) | 0.03 |
| (Missing) | 135 | 69 | 66 |  |
| Immunocompromised*^5^* | 269 (15%) | 89 (9.3%) | 180 (21%) | <0.001 |
| (Missing) | 137 | 69 | 68 |  |
| Systemic Immunosuppressive Therapy or Medications | 293 (16%) | 117 (12%) | 176 (20%) | <0.001 |
| (Missing) | 115 | 59 | 56 |  |
| *^1^* N= number of participants  *^2^* N (%) or median (Interquartile Range [IQR])  *^3^* p= p-value for staticial significance  *^4^* Pearson's Chi-squared test or Fisher’s exact test for categorical variables and Wilcoxon rank-sum test for continuous variables  *^5^* Immunocompromised (e.g., HIV infection, active cancer, solid organ transplant, hematopoietic stem cell transplant) | | | | |
