# Supplementary material for: Viral and host factors associated with SARS-CoV-2 disease severity in Georgia, USA
Source: PLoS One. 2025 Apr 1;20(4):e0317972. doi: 10.1371/journal.pone.0317972 (PMC11960886; doi:10.1371/journal.pone.0317972)
Supplement: S2 Table — (DOCX) [file pone.0317972.s004.docx]

| **Table S2. Circulation of Variants of Concern (VOC) and Variants of Interest (VOI) in Georgia, USA as measured by available sequences on GISAID** | | | | |
| --- | --- | --- | --- | --- |
| **VOC/VOI** | **Period variant circulated in GA***^1^* | **Date range of sequences obtained from GISAID** | **Sequences available on GISAID** | **Total sequences available from May 2021 to May 2022 (%)** |
| **Alpha** | 12/10/2020-08/26/2021 | 05/01/2021-08/26/2021 | 1,361 | 2.66 |
| **Beta** | 02/01/2021-05/25/2021 | 05/01/2021-05/25/2021 | 16 | 0.03 |
| **Delta** | 4/29/2021-03/10/2022 | 05/01/2021-03/10/2022 | 28,320 | 55.5 |
| **Gamma** | 03/05/2021-09/24/2021 | 05/01/2021-09/24/2021 | 185 | 0.36 |
| **Lamba** | 02/07/2021-08/05/2021 | 05/01/2021-08/05/2021 | 18 | 0.04 |
| **Mu** | 04/23/2021-09/14/2021 | 05/01/2021-09/14/2021 | 142 | 0.28 |
| **Omicron** | 11/30/2021-4/18/2023 | 11/30/2021-04/18/2023 | 20,759 | 40.6 |
|  | **Total VOC/VOI** | | **50,801** | **99.46** |
| **Total sequences available on GISAID from May 2021 to May 2022 from the state of Georgia** | | | **51,075** | **---** |
| *^1^*Period of circulation based on sequences available on GISAID including Emory Healthcare samples. | | | | |
