# Supplementary material for: Viral and host factors associated with SARS-CoV-2 disease severity in Georgia, USA
Source: PLoS One. 2025 Apr 1;20(4):e0317972. doi: 10.1371/journal.pone.0317972 (PMC11960886; doi:10.1371/journal.pone.0317972)
Supplement: S3 Table — (DOCX) [file pone.0317972.s005.docx]

| **Table S3. Unadjusted Multinomial Logistic Regression Models Testing the Association between Demographics, Underlying Health Conditions, and COVID-Related Characteristics with Disease Severity** | | | | | | | | | |
| --- | --- | --- | --- | --- | --- | --- | --- | --- | --- |
|  | **Moderate***^1^* | | | **Severe***^1^* | | | **Death***^1^* | | |
| **Variable** | **OR***^2^* | **95% CI***^2^* | **p***^3,4^* | **OR***^2^* | **95% CI***^2^* | **p***^3,4^* | **OR***^2^* | **95% CI***^2^* | **p***^3,4^* |
| **Unadjusted Models** | | | | | | | | | |
| **Demographics** | | | | | | | | | |
| ***Age*** | ***1.04*** | ***1.03, 1.05*** | ***<0.001*** | ***1.05*** | ***1.04, 1.05*** | ***<0.001*** | ***1.07*** | ***1.05, 1.09*** | ***<0.001*** |
| ***Male****^5^* | ***1.40*** | ***1.13, 1.74*** | ***<0.01*** | 1.30 | 0.95, 1.78 | 0.10 | 1.26 | 0.72, 2.22 | 0.40 |
| White*^6^* | 0.86 | 0.67, 1.10 | 0.20 | 1.27 | 0.91, 1.77 | 0.20 | 1.04 | 0.57, 1.91 | 0.90 |
| **Underlying Conditions** | | | | | | | | | |
| ***Chronic Lung Disease*** | ***2.07*** | ***1.59, 2.68*** | ***<0.001*** | ***1.95*** | ***1.35, 2.81*** | ***<0.001*** | ***2.65*** | ***1.44, 4.86*** | ***<0.01*** |
| ***Hypertension*** | ***2.94*** | ***2.35, 3.68*** | ***<0.001*** | ***4.19*** | ***2.98, 5.90*** | ***<0.001*** | ***11.4*** | ***5.09, 25.6*** | ***<0.001*** |
| ***Overweight*** | ***1.45*** | ***1.15, 1.83*** | ***<0.01*** | ***1.58*** | ***1.14, 2.19*** | ***0.01*** | ***2.02*** | ***1.13, 3.61*** | ***0.02*** |
| ***Cardiovascular Disease*** | ***2.89*** | ***2.28, 3.67*** | ***<0.001*** | ***3.46*** | ***2.48, 4.81*** | ***<0.001*** | ***4.80*** | ***2.70, 8.54*** | ***<0.001*** |
| ***Diabetes*** | ***2.04*** | ***1.58, 2.63*** | ***<0.001*** | ***3.21*** | ***2.29, 4.50*** | ***<0.001*** | ***4.35*** | ***2.43, 7.80*** | ***<0.001*** |
| ***Renal Disease*** | ***3.88*** | ***2.90, 5.19*** | ***<0.001*** | ***3.86*** | ***2.62, 5.67*** | ***<0.001*** | ***9.86*** | ***5.46, 17.8*** | ***<0.001*** |
| ***Liver Disease*** | *1.47* | *0.87, 2.50* | *0.20* | ***2.36*** | ***1.25, 4.45*** | ***<0.01*** | ***3.08*** | ***1.16, 8.21*** | ***0.02*** |
| ***Autoimmune Disease*** | ***1.60*** | ***1.01, 2.56*** | ***0.046*** | ***1.58*** | ***0.82, 3.03*** | ***0.20*** | ***2.45*** | ***0.93, 6.47*** | ***0.07*** |
| ***Immunocompromised*** | ***2.65*** | ***1.97, 3.58*** | ***<0.001*** | ***2.44*** | ***1.61, 3.69*** | ***<0.001*** | ***3.69*** | ***1.93, 7.09*** | ***<0.001*** |
| ***Systemic Immunosuppressive Therapy/Meds.*** | ***3.49*** | ***2.61, 4.67*** | ***<0.001*** | ***3.24*** | ***2.18, 4.81*** | ***<0.001*** | ***3.48*** | ***1.79, 6.78*** | ***<0.001*** |
| **Vaccination Status** |  |  |  |  |  |  |  |  |  |
| Unvaccinated | Ref | — |  | Ref | — |  | Ref | — |  |
| ***Vaccinated****^7^* | ***0.80*** | ***0.64, 1.01*** | ***0.07*** | 0.86 | 0.61, 1.21 | 0.40 | 1.00 | 0.54, 1.84 | >0.90 |
| ***Vaccinated & boosted****^7^* | ***2.28*** | ***1.52, 3.43*** | ***<0.001*** | ***3.24*** | ***1.95, 5.40*** | ***<0.001*** | ***3.69*** | ***1.58, 8.59*** | ***<0.01*** |
| **Days Since Most Recent Vaccination/Booster** | | | | | | | | | |
| Unvaccinated | — | — |  | — | — |  | — | — |  |
| Within past 90 Days*^7^* | 1.34 | 0.86, 2.08 | 0.20 | 1.68 | 0.93, 3.03 | 0.09 | 2.81 | 1.17, 6.79 | 0.02 |
| 91 - 180 Days Ago*^7^* | 0.98 | 0.74, 1.29 | 0.90 | 0.92 | 0.60, 1.40 | 0.70 | 1.14 | 0.55, 2.37 | 0.70 |
| 181 - 270 Days Ago*^7^* | 0.72 | 0.51, 1.01 | 0.06 | 0.74 | 0.45, 1.24 | 0.30 | 0.71 | 0.27, 1.89 | 0.50 |
| More than 270 Days Ago*^7^* | 1.18 | 0.72, 1.94 | 0.50 | 2.49 | 1.43, 4.36 | <0.01 | 1.91 | 0.64, 5.72 | 0.20 |
| **Lineage** | | | | | | | | | |
| Delta | Ref | — |  | Ref | — |  | Ref | — |  |
| ***Omicron****^8^* | ***1.51*** | ***1.09, 2.07*** | ***0.01*** | ***2.25*** | ***1.46, 3.48*** | ***<0.001*** | ***4.55*** | ***2.25, 9.20*** | ***<0.001*** |
| *^1^*^”^ Mild” is the Reference Category  *^2^* OR = Odds Ratio, CI = Confidence Interval, Ref = Reference Level  *^3^* p= p-value for staticial significance  *^4^* Pearson's Chi-squared test or Fisher’s exact test for categorical variables and Wilcoxon rank-sum test for continuous variables  *^5^* Compared to “Female”  *^6^* Compared to “Black”  *^7^* Compared to “Unvaccinated individuals  *^8^* Compared to “Delta” | | | | | | | | | |
